# Supplementary material for: Detection of ER Stress in iPSC-Derived Neurons Carrying the p.N370S Mutation in the GBA1 Gene
Source: Biomedicines. 2024 Mar 27;12(4):744. doi: 10.3390/biomedicines12040744 (PMC11047942; doi:10.3390/biomedicines12040744)
Supplement: Supplementary file 1 [file biomedicines-12-00744-s001.zip › biomedicines-2891209-supplementary.pdf]

Supplementary Materials

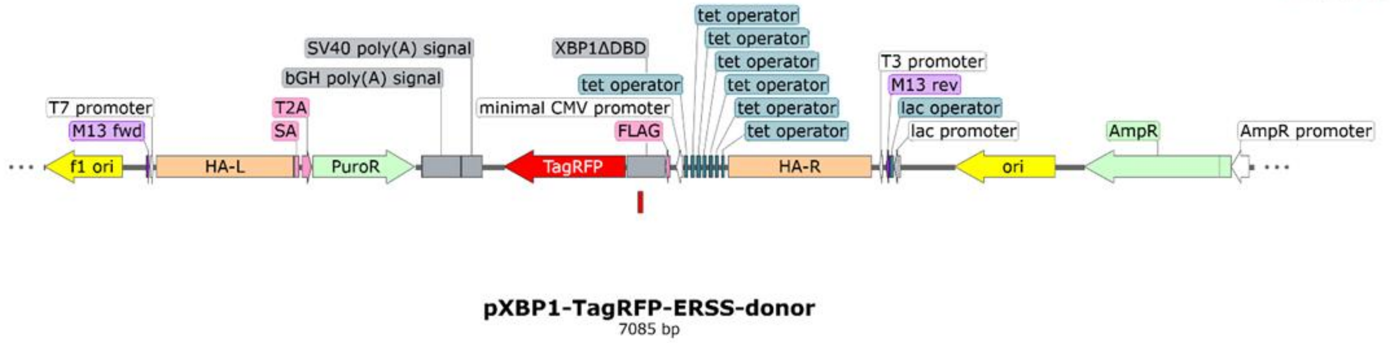

Figure S1 Plasmid map pXBP1-TagRFP-ERSS-donor.

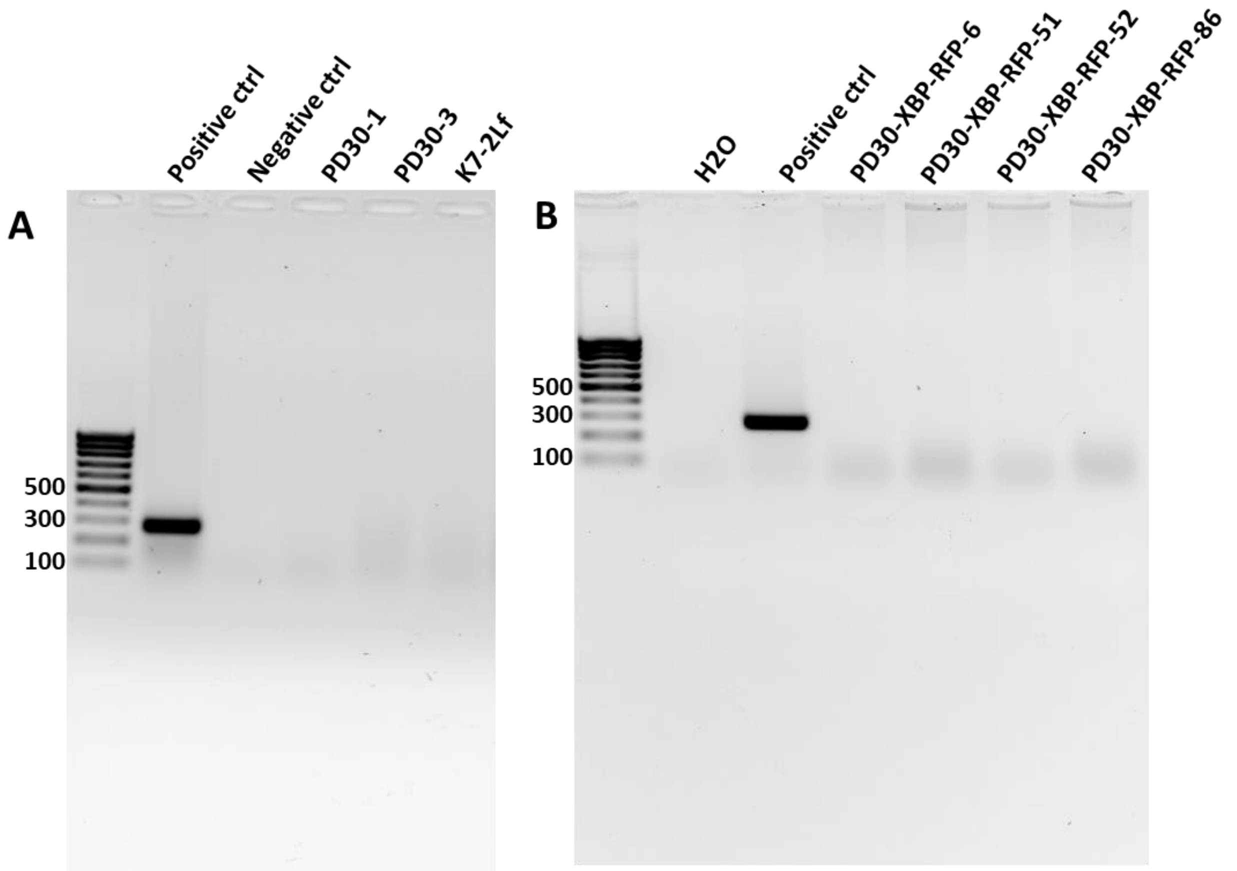

Figure S2 PCR test for mycoplasma of the iPSC lines. (A) iPSCs from a patient with Parkinson's disease associated with a mutation in the *GBA1* gene (PD30-1 and PD30-3) and iPSCs from a conditionally healthy individual (K7-2Lf). (B) Transgenic iPSCs carrying an ER stress biosensor XBP1-TagRFP (PD30-XBP-RFP-6, PD30-XBP-RFP-51, PD30-XBP-RFP-52 and PD30-XBP-RFP-86). DNA-marker Step100.

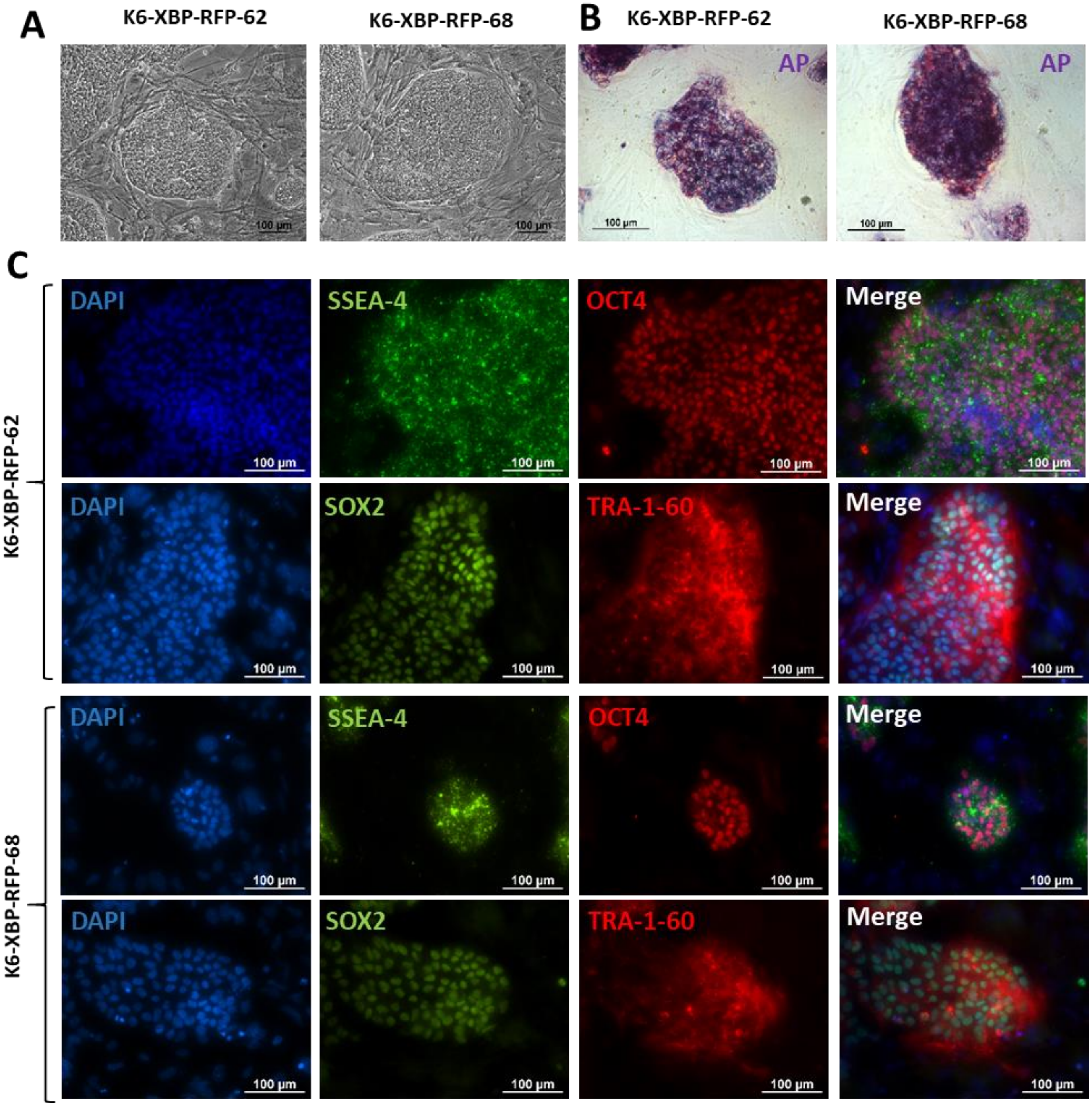

Figure S3 Characteristics of transgenic iPSCs carrying an ER stress biosensor XBP1-TagRFP (K6-XBP-RFP-62, K6-XBP-RFP-68). (A) Morphology of iPSC colonies. (B) Histochemical detection of alkaline phosphatase (AP). (C) Immunofluorescent staining for pluripotency markers OCT4 (red signal), SOX2 (green signal), SSEA-4 (green signal), TRA-1-60 (red signal). Nuclei are stained with DAPI (blue signal). All scale bars: 100 µm.

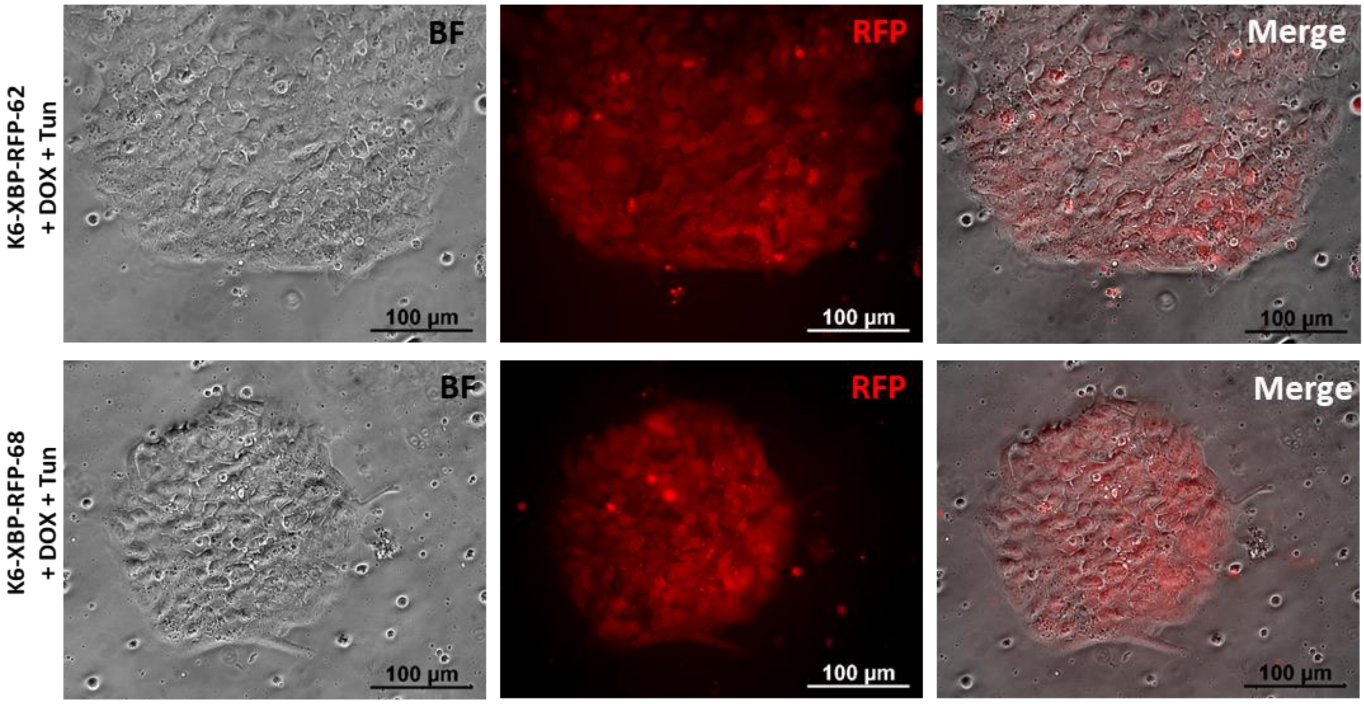

Figure S4 Visualization of the XBP1-TagRFP biosensor in two transgenic iPSC lines (K6-XBP-RFP-62 and K6-XBP-RFP-68). The iPSCs were cultured on the Matrigel extracellular matrix in Essential 8 medium. The operation of the transgene is ensured by the addition of doxycycline to the culture medium. ER stress is induced by the addition of tunicamycin to the culture medium.

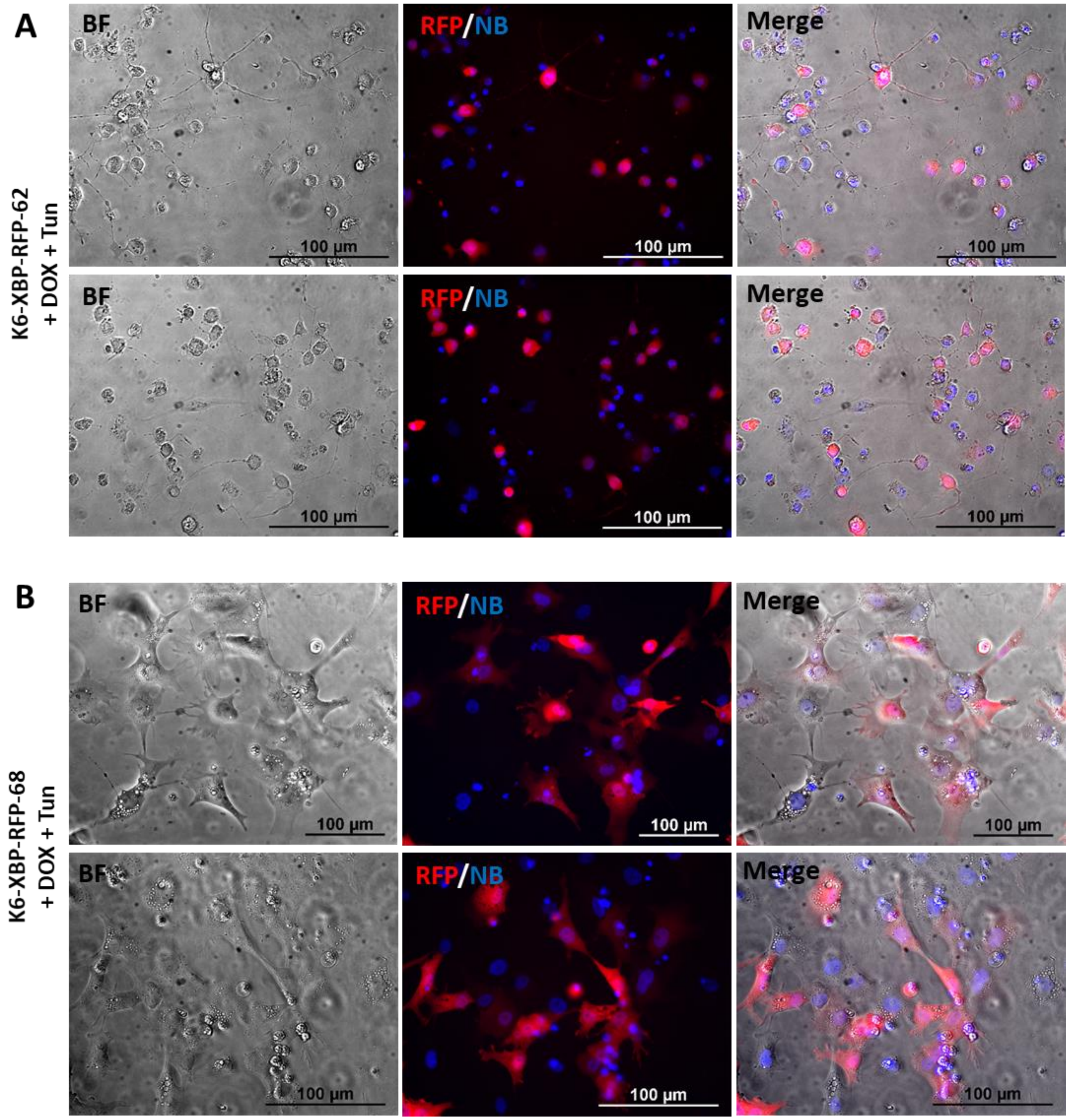

Figure S5 Visualization of the XBP1-TagRFP biosensor in neural derivatives from K6-XBP-RFP iPSCs. (A) Subclone K6-XBP-RFP-62 differentiated into DA-like neurons. (B) Subclone K6-XBP-RFP-68 differentiated into astrocyte-like derivatives. Intravital staining of nuclei (blue signal) – NucBlue (NB) (Thermo Fisher Scientific, Waltham, MA, USA). BF – bright field. All scale bars: 100 µm.
